# Novel optoelectronic devices based on single semiconductor nanowires (nanobelts)

**DOI:** 10.1186/1556-276X-7-218

**Published:** 2012-04-13

**Authors:** Yu Ye, Lun Dai, Lin Gan, Hu Meng, Yu Dai, Xuefeng Guo, Guogang Qin

**Affiliations:** 1 State Key Lab for Mesoscopic Physics and School of Physics, Peking University, Beijing, 100871, China; 2 College of Chemistry and Molecular Engineering, Peking University, Beijing, 100871, China

**Keywords:** Schottky junction, Graphene, Nanowires, Nanobelts, Optoelectronics

## Abstract

Semiconductor nanowires (NWs) or nanobelts (NBs) have attracted more and more attention due to their potential application in novel optoelectronic devices. In this review, we present our recent work on novel NB photodetectors, where a three-terminal metal–semiconductor field-effect transistor (MESFET) device structure was exploited. In contrast to the common two-terminal NB (NW) photodetectors, the MESFET-based photodetector can make a balance among overall performance parameters, which is desired for practical device applications. We also present our recent work on graphene nanoribbon/semiconductor NW (SNW) heterojunction light-emitting diodes (LEDs). Herein, by taking advantage of both graphene and SNWs, we have fabricated, for the first time, the graphene-based nano-LEDs. This achievement opens a new avenue for developing graphene-based nano-electroluminescence devices. Moreover, the novel graphene/SNW hybrid devices can also find use in other applications, such as high-sensitivity sensor and transparent flexible devices in the future.

## Review

### Introduction

Semiconductor single-crystalline nanowires (NWs) or nanobelts (NBs) can be grown on lattice mismatched substrates and constructed into devices with the bottom-up method on basically any substrates [1]. Hence, compared to the conventional ones, semiconductor NW- or NB-based devices have the advantage of versatility in both the material and the device structure. So far, various semiconductor NW- or NB-based nanodevices have been emerging continuously [2-4]. Developing novel high-performance nano-optoelectronic devices is not only important in diverse device applications, but also has significant meaning in exploring and realizing optoelectronic integration.

In this review, we present our research work on two types of novel optoelectronic devices based on semiconductor NWs (NBs). One is semiconductor NB metal–semiconductor field-effect transistor (MESFET)-based photodetectors [5]. In contrast to the common two-terminal single semiconductor NB (NW) photodetectors, the three-terminal NB MESFET-based photodetector can make a balance among overall performance parameters, which is desired for practical device applications. The other is novel multicolor light-emitting diodes (LEDs) based on graphene nanoribbon (GNR)/semiconductor nanowire (SNW) heterojunctions [6]. Herein, ZnO, CdS, and CdSe NWs were employed for demonstration. At forward biases, the GNR/SNW heterojunction LEDs emitted light from ultraviolet (380 nm) to red (705 nm), which were determined by the bandgaps of the involved SNWs. This work opens a new avenue for developing diverse graphene-based optoelectronic devices [7]. These two works may help to promote nano-optoelectronic integration in the future.

### Single CdS NB MESFET photodetector

Photodetectors, which convert light to electric signals, are essential elements in high-resolution imaging techniques and light-wave communication, as well as in future memory storage [8]. Single NB (NW) photodetectors may find applications as binary switches, light-wave communications, and optoelectronic circuits. So far, most of the reported single NB (NW) photodetectors are two-terminal devices [3, 8-17]. We summarize the key parameters of the three-terminal MESFET CdS NB photodetector and the reported two-terminal CdS NB (NW) photodetectors in Table 1. In general, for the two-terminal NB (NW) photodetectors, there exists a trade-off among the performance parameters, such as current responsivity (*R*_λ_), photoresponse ratio (*I*_light_/*I*_dark_), and photoresponse time (rise and fall times). For example, Golberg et al. reported ohmic contact-based single CdS NB photodetectors with ultrahigh *R*_λ_ (approximately 7.3 × 10^4^ A/W) and fast response time (approximately 20 μs of both rise and fall times); however, the *I*_light_/*I*_dark_ was quite low (approximately 6) [15]. Wang et al. reported Schottky contact-based NW photodetectors with a higher *I*_light_/*I*_dark_ (approximately183); however, the response time was not satisfying (approximately 320 ms of fall time) [17]. Compared to the reported two-terminal NB (NW) photodetectors, the MESFET-based photodetector can make a balance among these key parameters and have an overall improvement in the device performance.

**Table 1:** Comparison of the key parameters for the CdS NB (NW) photodetectors with different structures

**Materials**	**Structure**	**Rise; fall time**	**Responsivity (A/W)**	***I***_**light**_**/*I***_**dark**_	**Reference**
CdS NB	Ohmic contact	746; 794 μs	approximately 38	6.0 × 10^3^	[9]
CdS NB	Ohmic contact	approximately 20; approximately 20 μs	7.3 × 10^4^	6.0	[15]
CdS NW	Schottky contact	-; 320 ms	-	approximately 183.0	[17]
CdS NB	MESFET	137; 379 μs	approximately 2.0 × 10^2^	approximately 2.7 × 10^6^	This work

**Figure 1:**
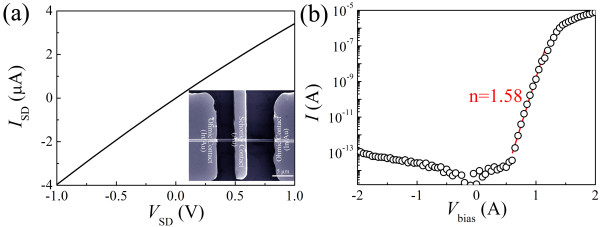
**Typical electrical transport properties of the single CdS NB MESFETs.** (**a**) The current–voltage (*I*-*V*) curve measured between the source and drain electrodes. Inset: a typical FESEM image of a single CdS NB MESFET-based photodetector. (**b**) The red straight line shows the fitting result with the equation $\text{ln}\left(I\right)=\frac{qV}{nkT}+\text{ln}\left(I_{0}\right)$ where *I*_*0*_ is the reverse saturation current, *q* is the electronic charge, *V* is the applied bias, *n* is the diode ideality factor, *k* is the Bolzmann constant, and *T* is the absolute temperature.

A field-emission scanning electron microscopy (FESEM) image of the as-fabricated photodetector was shown in the inset of Figure 1a. Two In/Au (10:100 nm) ohmic contact electrodes were defined on one single CdS NB, while one Au (100 nm) Schottky contact electrode was defined in between the ohmic electrodes across the CdS NB. We can see that the NB has a uniform width (500 nm) along the entire length (13 μm) between the two ohmic contacts.

**Figure 2:**
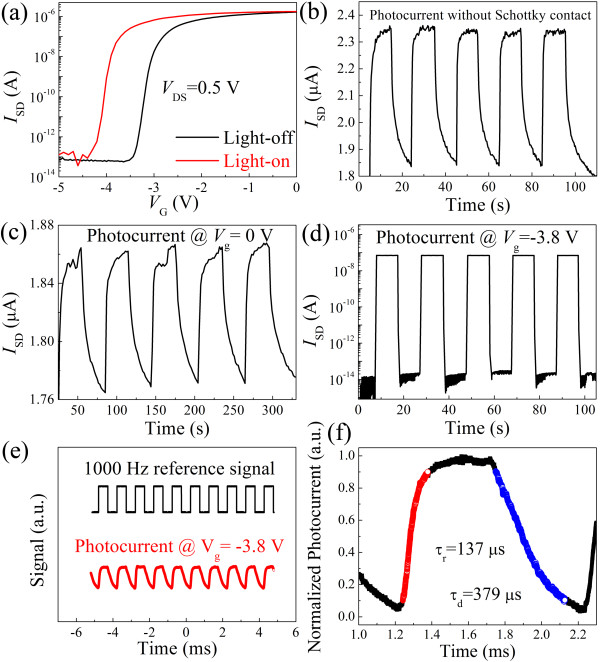
**The typical light response properties of the single CdS NB MESFET-based photodetectors.** (**a**) The transfer characteristics of a CdS NB MESFET-based photodetector measured in the dark (black line) and under illumination (red line). (**b**) On/off photocurrent response of the CdS NB without Schottky contact as a function of time. (**c**) On/off photocurrent response of the CdS NB MESFET-based photodetector with *V*_G_ = 0 V as a function of time on a linear scale. (**d**) On/off photocurrent response of the CdS NB MESFET-based photodetector with *V*_G_ = −3.8 V as a function of time on an exponential scale. (**e**) A transient response of the CdS NB MESFET-based photodetector (*V*_G_ = −3.8 V, *V*_DS_ = 0.5 V) along with a reference signal of the chopped light with a frequency of 1,000 Hz. (**f**) A close-up of the result shown in (e).

Typical electrical transport properties of the CdS NB MESFETs are shown in Figure 1. During the electrical transport measurements, the source electrodes were grounded. Figure 1a shows the *IV* curve measured between the source and drain electrodes. It shows a linear behavior, confirming the ohmic contacts between the In/Au electrodes and the CdS NB. Figure 1b shows the *IV* curve measured between the source and gate electrodes on an exponential scale. We can see a good Schottky contact rectification behavior between the Au electrode and the CdS NB. A rectification ratio of approximately 10^8^ is obtained when the voltage changes from +2 to −2 V. The turn-on voltage is around 1.25 V. The typical transfer characteristic curves of the CdS NB MESFETs with and without light illumination are depicted in Figure 2a. We can see that the *V*_th_ shifts from −2.9 to −3.8 V when the light is switched from off-state to on-state. This phenomenon can be understood as follows: there are two processes involved when the as-fabricated device is upon above-bandgap illumination. One is that the channel conductance increases due to the photon-generated electrons and holes; the other is that photon-generated electrons and holes at the Schottky junction are separated by the strong local electric field [16,18], which may reduce the electron–hole recombination rates and lower the barrier height [16-19]. Both processes will make the CdS NB channel more difficult to be depleted, and hence, the *V*_th_ shifts to a more negative value. The photocurrent response of a control device, a two-terminal CdS NB photodetector, is shown in Figure 2b. It has a small photoresponse ratio (*I*_light_/*I*_dark_ approximately 2.78) and a long decay tail (tens of seconds). The photocurrent response of the CdS NB MESFET measured at *V*_G_ = 0 V is shown in Figure 2c. We can see that the average dark current (light-off) and photocurrent (light-on) are about 1.77 and 1.86 μA, respectively, resulting in an *I*_light_/*I*_dark_ of approximately 1.05. Again, a long decay tail of tens of seconds can be observed.

Figure 2d shows the on/off photocurrent response of the CdS NB MESFET-based photodetector measured at *V*_G_ = −3.8 V, which is the threshold voltage of the MESFET under light illumination. We can see that the average dark current and photocurrent are about 26 fA and 70 nA, respectively, resulting in a *I*_light_/*I*_dark_ as high as approximately 2.7 × 10^6^. To the best of our knowledge, this is so far among the highest reported values for single NB (NW) photodetectors [3, 8-17]. In addition, the photoresponse processes (both rise and decay processes) are quite fast, which have exceeded the detection limit (0.3 s) of the measurement apparatus (Keithley 4200, Cleveland, OH, USA).

The *R*_λ_, defined as the photocurrent generated per unit power of incident light on the effective illuminated area of a photoconductor, and the external quantum efficiency (EQE), defined as the number of electrons detected per incident photon, are two critical parameters for photodetectors. The *R*_λ_ and EQE can be calculated with equations $R_{\text{λ}}=\frac{\Delta I}{P_{\text{λ}}S}$ and $\text{EQE}=\frac{hcR_{\text{λ}}}{e\lambda }$[11], respectively. Here, Δ*Ι* is the difference between the photocurrent and the dark current, *P*_λ_ is the light power density, *S* is the effective illuminated area, *h* is Planck's constant, *c* is the velocity of light, *e* is the electronic charge, and *λ* is the light wavelength. Using Δ*Ι* = 7.0 × 10^−8^ A (measured from Figure 2d), *P*_λ_ = 5.3 mW/cm^2^*S* = 500 nm × 13 μm (measured from the inset of Figure 1a), *λ* = 488 nm, the *R*_λ_ and EQE of the CdS NB MESFET photodetector can be estimated to be approximately 2.0 × 10^2^ A/W and 5.2 × 10^2^, respectively.

In order to further investigate the detailed photoresponse times of the single CdS NB MESFET photodetector, we employed a 200-MHz digital oscilloscope (Tektronix DPO2024, Beaverton, OR, USA) with a 10-MΩ impedance and an optical chopper working at a frequency of 1,000 Hz, as shown in Figure 2e. From the close-up of the measured result shown in Figure 2f, the rise timer, defined as the time needed for the photocurrent to increase from 10 % *i*_peak_ to 90 % *i*_peak_, is 137 μs and the decay timed, defined analogously, is 379 μs.

We attribute the overall high performance of our CdS NB MESFET-based photodetectors to the unique advantage of the MESFET structure. Compared to two-terminal photodetectors, there are two main advantages of the MESFET-based photodetectors. First, it has a much lower dark current because the applied negative gate voltage (in our case, the threshold voltage under illumination) helps to deplete the channel carriers. Second, this gate depletion effect will also cause a fast current recovery when the light is turned off. Consequently, the decay tail, which is normally observed in a two-terminal photodetectors, is suppressed in the MESFET-based photodetectors.

### Multicolor GNR/SNW heterojunction LEDs

By taking advantage of both graphene and SNWs, we have fabricated, for the first time, the graphene-based nano-LED [6]. This achievement opens a new avenue for developing graphene-based nano-electroluminescence devices. Moreover, the novel graphene/SNW hybrid devices can also find use in other applications, such as high-sensitivity sensor and transparent flexible devices in the future.

Both the n-type NWs [20-22] and the graphene [23] used in this work were synthesized via the CVD method. Before device fabrication, the graphenes were transferred by the stamp method with the help of polymethyl methacrylate [24] to Si/300-nm SiO_2_ substrates for Raman and electrical property characterizations, to quartz substrates for transparency characterization, and transferred to carbon-coated grids for high-resolution transmission electron microscopy (HRTEM) characterization (Tecnai F30, FEI, Eindhoven, The Netherlands). Their electrical properties were measured by a Hall effect measurement system (Accent HL5500, York, England).

**Figure 3:**
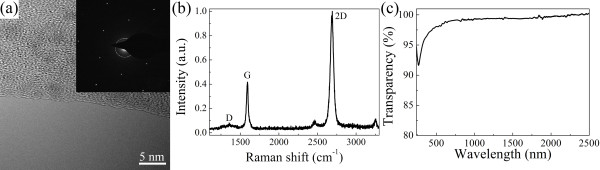
**Properties of as-synthesized graphene.** (**a**) Typical HRTEM image of an as-synthesized graphene, indicating the formation of monolayer graphene. Inset: selected area electron diffraction pattern of the graphene. (**b**) Raman spectrum of an as-synthesized graphene on a Si/300-nm SiO_2_ substrate. (**c**) The transparency spectrum of the graphene on a quartz substrate.

The HRTEM, Raman, and transparency characterization results for the as-synthesized graphenes (Figure 3) demonstrate that the graphenes have high quality, monolayer, and high transparency. The typical sheet resistance, hole concentration, and hole mobility of the graphenes are about 345 Ω/sq, 1.84 × 10^14^ cm^–2^, and 98.6 cm^2^/V·s, respectively.

**Figure 4:**
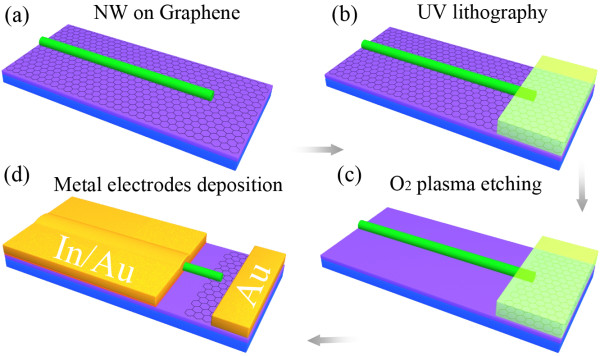
**Schematic illustration of the fabrication processes of a GNR/SNW heterojunction LED.** (**a**) The as-synthesized large-scale graphene was transferred to a Si/SiO_2_ substrate. After that, SNW suspension was dropped on the graphene. (**b**) A photoresist pad was patterned to cover one end of a SNW by UV lithography and development processes. (**c**) Oxygen plasma etching was used to remove the exposed graphene. After that, the GNR formed under the SNW. (**d**) After removing the photoresist, In/Au and Au ohmic contact electrodes to SNW and graphene pad were defined, respectively. It is worth noting that because an undercut was formed during the oxygen plasma etching process (Figure 4c) [6, 27], the In/Au electrode on the SNW will not contact with the GNR beneath.

**Figure 5:**
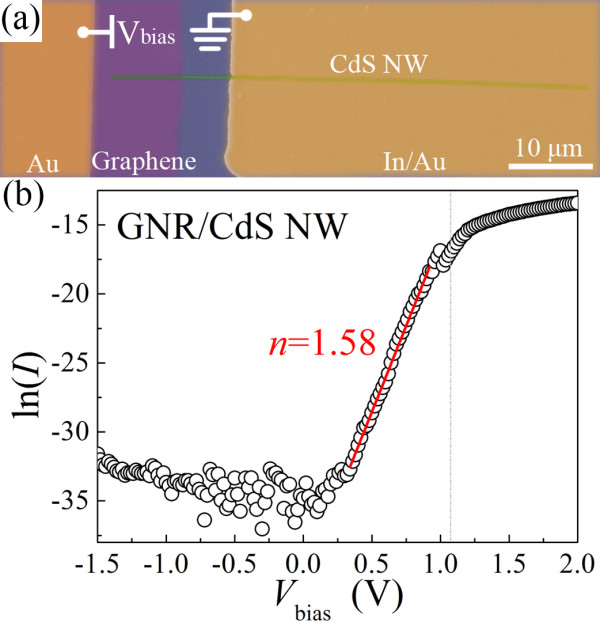
**FESEM image and room-temperature*I*-*V*characteristic of as-fabricated GNR/CdS NW heterojunction LED.** (**a**) FESEM image of an as-fabricated GNR/CdS NW heterojunction LED. (**b**) Room-temperature *I*-*V* characteristic of the LED in (a) on a semilog scale. The red straight line shows the fitting result of the *I*-*V* curve by the equation $\text{ln}\left(I\right)=\frac{qV}{nkT}+\text{ln}\left(I_{0}\right)$.

The fabrication processes of a GNR/SNW heterojunction LED are shown in Figure 4. Figure 5a shows an FESEM image of an as-fabricated GNR/CdS NW heterojunction LED. The *IV* curve (Figure 5b) of the LED shows an excellent rectification characteristic. An on/off current ratio of approximately 3.4 × 10^7^ can be obtained when the voltage changes from +1.5 to −1.5 V. The turn-on voltage is around 1.1 V. In view of the high conductivity and near-zero bandgap characteristics of the GNR [25], the heterojunction structure of the GNR/CdS NW can be considered approximately as a metal–semiconductor contact of the Schottky model [26]. We can deduce that the diode ideality factor *n* = 1.58. Note that the GNR/ZnO NW and GNR/CdSe NW heterojunctions show similar rectification characteristics as described above, with the turn-on voltages to be about 0.7 and 1.2 V, respectively.

**Figure 6:**
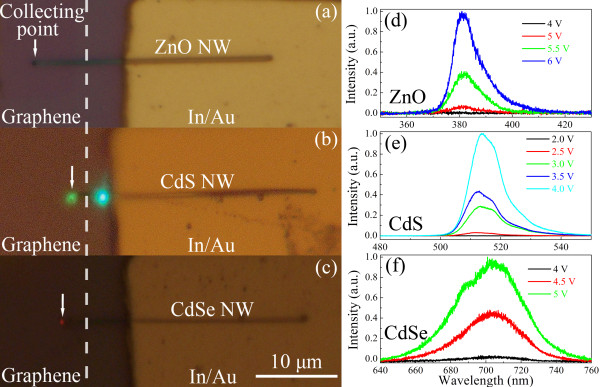
**Optical images and room-temperature EL spectra of the GNR/SNW heterojunction LEDs.** (**a, b, c**) The optical images of the GNR/SNW (ZnO, CdS, CdSe, respectively) heterojunction LEDs at a forward bias of 5 V. Dashed lines were used to demarcate the graphenes from the substrates. White arrows: the light collecting points during the EL measurements. (**d, e, f**) Room-temperature EL spectra for GNR/SNW (ZnO, CdS, CdSe, respectively) heterojunction LEDs at various forward biases.

Figure 6a,b,c shows the electroluminescence (EL) images (Olympus BX51M, Shinjuku-ku, Japan) of the GNR/SNW (ZnO, CdS, CdSe, respectively) heterojunction LEDs at a forward bias of 5 V. Except for the ZnO NW case (where the emitting light is invisible ultraviolet light) in Figure 6a, strong emitting light spots can be seen clearly with naked eyes at the exposed ends of the NWs. For the CdS NW case (Figure 6b), we can see another glaring light spot on the NW. This may be due to the scattering from the defect or adhered particle on the CdS NW [28]. Figure 6d,e,f shows the room-temperature EL spectra measured at various forward biases for the GNR/SNW heterojunction LEDs, where the SNWs are ZnO, CdS, and CdSe NWs, respectively. For all the LEDs, EL intensities increase with the forward biases. The peak wavelength of each EL spectrum (380, 513, and 705 nm, respectively, from (d) to (f)) coincides with the band-edge emission of the SNW involved. This indicates that the radiative recombination of electrons and holes occurs in the SNWs.

**Figure 7:**
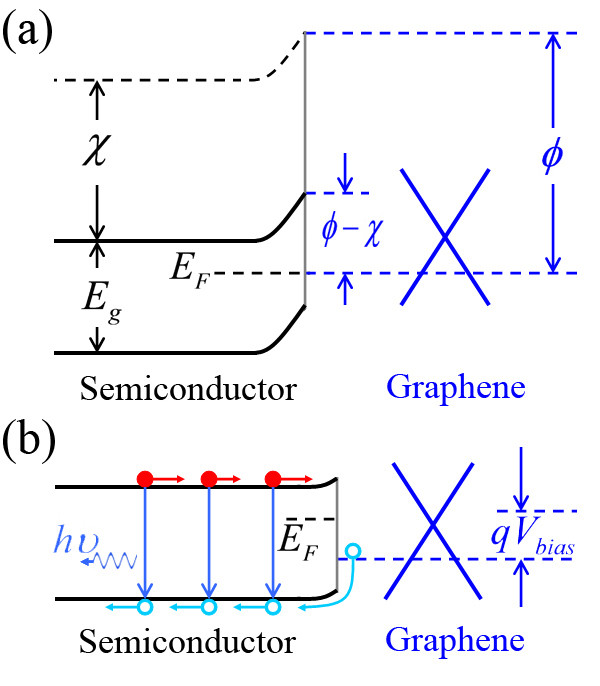
**Schematic illustration of the energy band diagrams of a graphene/semiconductor heterojunction.** (**a**) The thermal equilibrium energy band diagram. (**b**) The energy band diagram of the heterojunction under a forward bias. *Φ*: the work function of graphene; *χ*: the electron affinity of the semiconductor.

We can qualitatively understand the mechanism of the light emitting for the GNR/SNW heterojunction LEDs by studying the energy band diagrams. Figure 7a shows the thermal equilibrium energy band diagram of a graphene/n-type semiconductor structure, where the work function of graphene is *Φ*, and the electron affinity of the semiconductor is *χ*. *E*_g_ and *E*_F_ correspond, respectively, to the bandgap and the Fermi level of the semiconductor. It is worth noting that because the graphene used in this work has a very high conductivity and can be taken as a metal, the graphene/SNW heterostructure herein can be taken as a kind of Schottky junction. At the thermal equilibrium contacting state, the energy band of the semiconductor will bend upward at the graphene/semiconductor interface due to the difference between their work functions, and the Fermi levels at the two sides are brought into coincidence. Under a forward bias (i.e., a positive bias on graphene), the built-in potential is lowered. Therefore, more electrons will flow from n-type semiconductor to graphene, and simultaneously, more holes will flow from graphene to n-type semiconductor. Herein, the injected holes have a higher radiative recombination with the electrons in the SNW (the direct bandgap semiconductor). Accordingly, the EL spectra are determined mainly by the band-edge emission of the SNWs.

It is worth noting that the GNR/NW structure has clear advantage over the conventional Schottky structure. For comparison, we have fabricated various metal/SNW Schottky structures, where the NWs used are identical to those reported in this work. Unfortunately, no EL can be observed in these structures. We attribute this to the well-known luminescence quenching effect caused by the involved metal [29]. Moreover, in our face-to-face contact LED, the active region, where the radiative recombination occurs, is larger and the series resistance is smaller, compared to the crossed NWs or NW/Si pad heterojunction structures [20, 30, 31]. These merits may benefit high-efficiency EL and even electrically driven laser in the future.

## Conclusion

We review two types of novel nano-optoelectronic devices developed in our group recently. One is the photodetector, which converts light to electric signals. Our MESFET-based photodetectors have ultrahigh *I*_light_/*I*_dark_ (approximately 2.7 × 10^6^) and fast response (rise time, approximately 137 μs; decay time, approximately 379 μs) simultaneously. The other is LED, which converts electric power to light. At forward biases, our novel GNR/SNW heterojunction LEDs emitted light with wavelengths varying from ultraviolet (380 nm) to red (705 nm), which were determined by the bandgaps of the involved SNWs. These two types of nano-optoelectronic devices may find diverse applications in future nano-optoelectronic integration.

## Competing interests

The authors declare that they have no competing interests.

## Authors' contributions

YY carried out the device fabrications, participated in the statistical measurements, and drafted the manuscript. LD and GQ participated in the instruction, discussion, and manuscript revision. LG and XG synthesized the graphene. HM participated in the device design. YD synthesized the CdSe NWs. All authors have read and approved the final manuscript.
